# Genome-wide identification, characterization, and expression analysis of the *small auxin-up RNA* gene family during zygotic and somatic embryo maturation of the cacao tree (*Theobroma cacao*)

**DOI:** 10.1186/s44342-024-00003-6

**Published:** 2024-05-28

**Authors:** Ngoc Thi Bich Chu, Man Thi Le, Hong Viet La, Quynh Thi Ngoc Le, Thao Duc Le, Huyen Thi Thanh Tran, Lan Thi Mai Tran, Chi Toan Le, Dung Viet Nguyen, Phi Bang Cao, Ha Duc Chu

**Affiliations:** 1 Faculty of Natural Sciences, Hung Vuong University, Viet Tri City, Phu Tho Province 35000 Vietnam; 2 Institute of Research and Application, Hanoi Pedagogical University 2, Phuc Yen City, Vinh Phuc Province 280000 Vietnam; 3 Department of Biotechnology, Thuyloi University, Hanoi City, 116830 Vietnam; 4 Agricultural Genetics Institute, Vietnam Academy of Agricultural Sciences, Hanoi City, 143330 Vietnam; 5 Faculty of Biology, Hanoi National University of Education, Xuan Thuy Road, Cau Giay District, Hanoi City, 122300 Vietnam; 6 Faculty of Biology and Agricultural Engineering, Hanoi Pedagogical University 2, Phuc Yen City, Vinh Phuc Province 280000 Vietnam; 7 Thanh Thuy Junior High School, Thanh Thuy District, Phu Tho Province 35850 Vietnam; 8 Faculty of Agricultural Technology, University of Engineering and Technology, Vietnam National University Hanoi, Xuan Thuy Road, Cau Giay District, Hanoi City, 122300 Vietnam

**Keywords:** Small auxin-up RNA, Cacao, Characteristic, Expression pattern, Genome-wide

## Abstract

Small auxin-up RNA (SAUR) proteins were known as a large family that supposedly participated in various biological processes in higher plant species. However, the SAUR family has been still not explored in cacao (*Theobroma cacao* L.), one of the most important industrial trees. The present work, as an in silico study, revealed comprehensive aspects of the structure, phylogeny, and expression of *TcSAUR* gene family in cacao. A total of 90 members of the *TcSAUR* gene family have been identified and annotated in the cacao genome. According to the physic-chemical features analysis, all TcSAUR proteins exhibited slightly similar characteristics. Phylogenetic analysis showed that these TcSAUR proteins could be categorized into seven distinct groups, with 10 sub-groups. Our results suggested that tandemly duplication events, segmental duplication events, and whole genome duplication events might be important in the growth of the *TcSAUR* gene family in cacao. By re-analyzing the available transcriptome databases, we found that a number of *TcSAUR* genes were exclusively expressed during the zygotic embryogenesis and somatic embryogenesis. Taken together, our study will be valuable to further functional characterizations of candidate *TcSAUR* genes for the genetic engineering of cacao.

## Introduction

Cacao (*Theobroma cacao* L.) has been known as one of the most critical industrial crops globally, which belongs to the family Malvaceae. Originating from the Central and South America [1, 2], cacao has grown up to at least fifty nations located in the humid tropics. As an excellent source of essential nutrients, minerals and antioxidants, cacao beans have been used for chocolate production, confectionery, and cosmetics [3, 4]. However, climate change, especially biotic and abiotic stresses could threaten cocoa production [5, 6].

To address the issues caused by adverse environmental conditions, various studies have concentrated on the functions of gene families [7–10], because understanding the roles of these functional and regulatory genes may open up the possibility of developing new climate change-adapted lines through genetic engineering. Of our interest, the small auxin-up RNA (SAUR) proteins serve as the largest sub-group of the expansive auxin response factor gene family in higher plant species. Particularly, the expression of *SAUR* genes is elicited rapidly and transiently by auxin, thus playing crucial roles in regulating plant growth, development, and responses to environmental stresses [11]. To gain an insight into their functions, previous studies have been performed to analyze the SAUR families in many important crop species, including potato (*Solanum tuberosum*) and tomato (*Solanum lycopersicum*) [12], watermelon (*Citrullus lanatus*) [13], cotton (*Gossypium* spp.) [14], moso bamboo (*Phyllostachys edulis*) [15], poplar (*Populus trichocarpa*) [16], grape (*Vitis vinifera*) [17], apple (*Malus domestica*) [18], coffea (*Coffea canephora*) [19], Chinese white pear (*Pyrus bretschneideri*) [20], melon (*Cucumis melo*) [21], loquat (*Eriobotrya japonica*) [22], wax gourd (*Benincasa hispida*) [23], peanut (*Arachis hypogaea*) [24], pineapple (*Ananas comosus*) [25], foxtail millet (*Setaria italica*) [26], cucumber (*Cucumis sativus*) [27], and longan (*Dimocarpus longan*) [28]. However, this important gene family in cacao has not been recorded, even though the assembly of cacao was released [29].

Thus, the aim of this present study was to systematically identify, annotate and characterize the SAUR family in cacao. Firstly, all putative members of the SAUR family have been screened and validated in the cacao assembly. By using various web-based tools, the general features of the proteins and genes were then explored. We then constructed an unrooted phylogenetic tree of the SAUR proteins and predicted the duplication events in the *SAUR* gene family. Finally, we re-analyzed the previous transcriptome database to investigate the expression levels of the *SAUR* genes in various tissues during zygotic embryogenesis and somatic embryogenesis.

## Materials and methods

### Identification of the SAUR genes in cacao

In order to identify the SAUR family members from cacao genomes, the whole genome and proteome data of *T. cacao* cultivar “B97-61/B2” (NCBI RefSeq assembly: GCF_000208745.1, date of release: Jul 9, 2016) were downloaded from the NCBI [29]. The hidden Markov model profile of the conservative functional domain of SAUR (Pfam accession: PF02519) [11] was obtained from the Pfam database [30]. All protein sequences were then screened against the cacao proteomes [29] to obtain the potential members of the *SAUR* gene family. The full-length protein sequence, genomic DNA sequence, and coding DNA sequence of each member of the SAUR family in cacao were obtained for subsequent analysis.

### Prediction of the SAUR protein characteristics in cacao

The full-length amino acid sequences of SAUR proteins in cacao were used as seed sequences for a search in the Expasy Protparam [31, 32] as previously guided [9, 33–36]. Particularly, the SAUR protein’s common features, including protein length, isoelectric point (pI), molecular weight (mW), aliphatic index (AI), and grand average of hydropathicity (GRAVY) were estimated.

### Construction of the phylogenetic tree of the SAUR proteins in cacao

The full-length amino acid sequences of SAUR proteins in cacao were used to generate an unrooted phylogenetic tree as previously guided [9, 33–36]. Firstly, the ClustalW software [37, 38] was used for the multisequence alignment of the SAUR proteins in cassava. Additionally, all members of the SAUR families from *Arabidopsis thaliana* [39, 40] and coffea [19] were also downloaded for other trees. Results were then imported into the Molecular Evolutionary Genetics Analysis (MEGA) software [41] for constructing an unrooted phylogenetic tree. A maximum likelihood estimation with default settings was applied as the model selection parameter. Finally, the Adobe Illustrator software was used to edit and visualize the resultant tree.

### Prediction of gene duplication of the SAUR genes in cacao

The duplicated events that occurred in the *SAUR* gene family in cacao were predicted based on the MEGA-based phylogenetic tree as previously described [9, 33–36]. Particularly, SAUR members in the same clade with high bootstrap values were assigned as duplicated pair. The criteria of sharing more than 70% identity were utilized for describing a duplicated gene pair. A duplicated pair was defined as a tandem duplication event if these genes are located next to each other on the same chromosome within a 100-kb distance, while a segmental duplication event referred to duplications of DNA segments that range in size from 1 to 200 kb and occur in the same chromosome [42]. Additionally, a duplicated pair resulting from a whole genome duplication event was known that duplicated genes were distributed in different chromosomes [42].

### Exon/intron structural analysis of the SAUR genes in cacao

Gene exon–intron structure characteristics of genes encoding the SAUR proteins in cacao were analyzed as previously guided [9, 33–36]. Specifically, the genomic DNA sequence and coding DNA sequence of each gene encoding SAUR protein in cacao were used to align in the Gene Structure Display Server [43]. The order of the SAUR proteins in cacao obtained from the phylogenetic tree was then applied to visualize the gene structures. We then used the Adobe Illustrator software to edit the figure.

### Transcriptome analysis of the SAUR genes in cacao

The expression profiles of the *SAUR* genes were analyzed based on the published transcriptome atlas available in the NCBI Gene Expression Omnibus [44] as previously described [9, 33–36]. We used the GSE55476 dataset to assess the expression levels of the *SAUR* genes in six tissue types and stages of embryogenesis [45]. Particularly, zygotic embryo tissues at 14 (T-ZE), 16 (EF-ZE), 18 (LF-ZE), and 20 weeks after pollination (M-ZE) and somatic embryo tissues harvested in the whole late torpedo stage (LT-SE) and cotyledon tissues from mature somatic embryos (M-SE) were extracted to prepare the library [45]. The genome-wide expression of the *SAUR* genes was visualized in R script [46]. The expression levels are described by a color bar that changes from green to red.

## Results and discussion

### Identification and annotation of TcSAUR genes in cacao

**Fig. 1:**
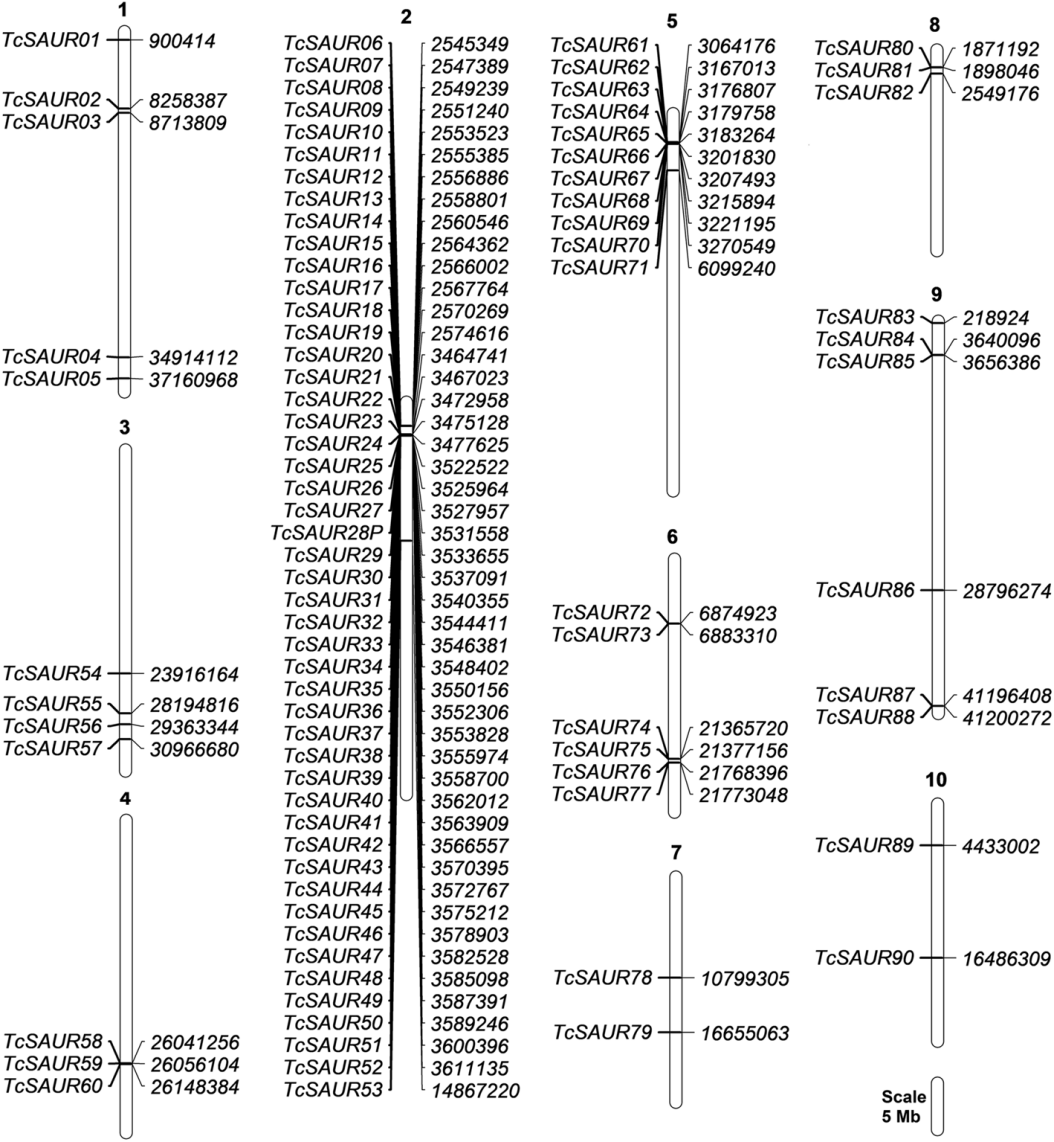
Physical distribution of the *SAUR* gene family in cacao genome

To identify all the putative *SAUR* genes in cacao, the seed sequence of the SAUR domain [11] was used to search against the proteome of cacao [29]. As a result, a total of 90 *SAUR* genes were identified and well-annotated in the genome of cacao (Table 1). Based on the order of occurrence in the cacao genome, all putative members of the SAUR family in cacao were defined from TcSAUR01 to TcSAUR90, with “Tc” and “SAUR” abbreviated for the scientific name of cacao (*Theobroma cacao*) and the full name of the protein (small auxin-up RNA) (Table 1, Fig. 1). It has been realized that all putative *TcSAUR* genes were localized in the cacao genome with an uneven ratio. Interestingly, the chromosomal distributions of the *SAUR* gene family in the genomes of other higher plant species, such as coffea [19], melon [21], and wax gourd [23] also confirmed our finding.

**Table 1:** Physical and chemical properties of the SAUR family in cacao

#	SAUR members	Phytozome accession	NCBI accession	Size	mW	pI	GRAVY	AI
1	TcSAUR01	Thecc1EG046673	XP_007046722.2	104	11.87	8.62	− 0.33	82.50
2	TcSAUR02	Thecc1EG001616	EOX92717.1	102	12.24	6.92	− 0.81	75.49
3	TcSAUR03	Thecc1EG001682	EOX92812.1	80	9.15	7.79	− 0.51	65.75
4	TcSAUR04	Thecc1EG005070	XP_017984353.1	115	13.39	7.90	− 0.51	83.83
5	TcSAUR05	Thecc1EG005545	EOX96266.1	167	19.09	10.44	− 0.54	74.67
6	TcSAUR06	Thecc1EG006512	XP_017970663.1	150	16.87	9.88	− 0.18	90.33
7	TcSAUR07	Thecc1EG006513	EOX97518.1	143	16.09	9.66	− 0.12	90.70
8	TcSAUR08	Thecc1EG006514	nd	149	16.91	9.12	− 0.14	98.12
9	TcSAUR09	nd	EOX97519.1	137	15.47	10.06	− 0.15	84.67
10	TcSAUR10	Thecc1EG006515	EOX97520.1	136	15.49	10.09	− 0.07	95.37
11	TcSAUR11	Thecc1EG006516	EOX97521.1	71	8.09	10.60	− 0.56	65.92
12	TcSAUR12	Thecc1EG006517	XP_017970652.1	140	15.71	8.61	− 0.26	80.93
13	TcSAUR13	nd	XP_017970637.1	148	16.72	9.24	− 0.07	97.50
14	TcSAUR14	Thecc1EG006518	XP_007041692.1	147	16.81	8.99	− 0.16	89.59
16	TcSAUR15	Thecc1EG006519	EOX97524.1	149	16.89	8.69	− 0.17	88.99
17	TcSAUR16	Thecc1EG006519	XP_017971984.1	149	16.69	9.69	− 0.20	89.73
17	TcSAUR17	Thecc1EG006521	EOX97526.1	149	16.55	9.40	− 0.15	87.05
18	TcSAUR18	Thecc1EG006522	EOX97527.1	151	16.83	9.33	0.09	101.99
19	TcSAUR19	Thecc1EG006523	EOX97528.1	148	16.63	8.76	− 0.12	85.68
20	TcSAUR20	Thecc1EG006722	EOX97790.1	94	10.58	6.96	− 0.14	100.53
21	TcSAUR21	Thecc1EG006725	XP_017970221.1	97	10.89	9.36	− 0.31	83.40
22	TcSAUR22	Thecc1EG006726	EOX97793.1	144	16.31	9.65	− 0.47	79.79
23	TcSAUR23	Thecc1EG006727	XP_017970327.1	106	12.07	8.53	− 0.31	80.00
24	TcSAUR24	Thecc1EG006728	EOX97795.1	103	11.36	6.25	− 0.09	78.64
25	TcSAUR25	Thecc1EG006733	XP_007041969.1	101	11.11	8.71	− 0.06	95.64
26	TcSAUR26	Thecc1EG006734	XP_007041970.1	93	10.51	6.39	0.14	102.58
27	TcSAUR27	Thecc1EG006735	EOX97802.1	95	10.56	8.54	0.01	88.32
28	TcSAUR28P	Thecc1EG006736	nd	93	10.52	6.90	− 0.04	92.15
29	TcSAUR29	Thecc1EG006737	XP_017970328.1	95	10.62	7.69	− 0.11	79.05
30	TcSAUR30	Thecc1EG006739	EOX97806.1	94	10.61	7.94	− 0.07	88.09
31	TcSAUR31	Thecc1EG006738	EOX97805.1	95	10.62	7.80	− 0.12	86.21
32	TcSAUR32	Thecc1EG006740	EOX97807.1	95	10.69	7.93	− 0.03	90.21
33	TcSAUR33	Thecc1EG006741	EOX97808.1	95	10.66	7.79	− 0.18	78.00
34	TcSAUR34	Thecc1EG006742	XP_017971161.1	93	10.41	6.55	0.01	99.57
35	TcSAUR35	Thecc1EG006742	XP_017971162.1	79	8.92	6.55	− 0.10	93.67
36	TcSAUR36	Thecc1EG006743	EOX97810.1	60	6.56	4.04	− 0.11	75.00
37	TcSAUR37	nd	XP_017971148.1	98	11.06	9.36	− 0.01	90.51
38	TcSAUR38	Thecc1EG006744	EOX97811.1	93	10.04	9.65	0.12	88.06
39	TcSAUR39	Thecc1EG006745	EOX97812.1	79	8.97	5.69	− 0.13	93.67
40	TcSAUR40	Thecc1EG006746	XP_017971149.1	134	15.52	8.93	0.03	82.16
41	TcSAUR41	Thecc1EG006746	XP_017971151.1	96	10.57	9.36	− 0.01	80.21
42	TcSAUR42	Thecc1EG006747	XP_017971152.1	79	8.98	7.88	− 0.08	92.41
43	TcSAUR43	Thecc1EG006747	XP_017971150.1	98	11.03	9.89	− 0.06	92.45
44	TcSAUR44	Thecc1EG006748	EOX97815.1	96	10.40	9.30	0.10	88.33
45	TcSAUR45	Thecc1EG006750	EOX97817.1	79	8.95	9.30	− 0.06	92.41
46	TcSAUR46	Thecc1EG006751	EOX97818.1	98	11.12	8.93	− 0.13	88.47
47	TcSAUR47	Thecc1EG006749	XP_017970146.1	96	10.47	9.36	− 0.03	77.19
48	TcSAUR48	Thecc1EG006752	EOX97819.1	98	11.08	7.81	− 0.11	85.51
49	TcSAUR49	Thecc1EG006753	EOX97820.1	100	11.19	7.93	0.02	89.70
50	TcSAUR50	Thecc1EG006754	XP_007041990.1	96	10.80	9.14	− 0.16	87.40
51	TcSAUR51	Thecc1EG006757	XP_017970560.1	104	11.88	8.51	− 0.25	87.12
52	TcSAUR52	Thecc1EG006758	EOX97827.1	160	18.01	10.03	− 0.33	79.75
53	TcSAUR53	Thecc1EG008557	XP_007043889.1	154	17.50	9.60	− 0.50	75.32
54	TcSAUR54	Thecc1EG014696	EOY22561.1	180	20.35	9.45	− 0.48	71.44
55	TcSAUR55	Thecc1EG015490	EOY23675.1	122	14.71	6.75	− 0.81	71.80
56	TcSAUR56	Thecc1EG015731	XP_017972364.1	124	14.62	5.20	− 0.26	97.34
57	TcSAUR57	Thecc1EG016036	EOY24431.1	163	18.95	10.53	− 0.46	81.84
58	TcSAUR58	Thecc1EG019987	EOY04815.1	104	11.73	9.64	− 0.58	75.10
59	TcSAUR59	Thecc1EG019992	EOY04818.1	104	11.83	9.63	− 0.50	79.71
60	TcSAUR60	Thecc1EG020007	XP_007033912.1	123	14.09	7.73	− 0.49	84.07
61	TcSAUR61	Thecc1EG022186	XP_017976323.1	123	14.10	8.79	− 0.23	82.36
62	TcSAUR62	Thecc1EG022211	nd	121	14.12	9.44	− 0.36	78.18
63	TcSAUR63	Thecc1EG022215	EOY07899.1	132	15.30	9.10	− 0.44	89.32
64	TcSAUR64	Thecc1EG022216	EOY07900.1	138	15.61	5.92	− 0.38	72.17
65	TcSAUR65	Thecc1EG022217	EOY07901.1	128	14.66	4.89	− 0.36	81.48
66	TcSAUR66	Thecc1EG022219	XP_017976773.1	128	14.71	8.44	− 0.10	107.97
67	TcSAUR67	Thecc1EG022220	EOY07903.1	164	18.08	4.68	0.07	90.91
68	TcSAUR68	Thecc1EG022221	EOY07904.1	116	13.32	4.73	− 0.43	78.19
69	TcSAUR69	Thecc1EG022222	EOY07905.1	129	14.33	4.41	− 0.36	77.05
70	TcSAUR70	Thecc1EG022229	EOY07909.1	99	11.15	9.41	− 0.17	99.49
71	TcSAUR71	Thecc1EG022627	XP_007027790.1	125	14.17	5.91	− 0.21	73.92
72	TcSAUR72	Thecc1EG027747	EOY26270.1	159	18.13	9.13	− 0.48	74.15
73	TcSAUR73	Thecc1EG027748	EOY26271.1	173	19.35	9.17	− 0.34	78.90
74	TcSAUR74	Thecc1EG029215	XP_007024729.1	101	11.51	9.94	− 0.27	80.10
75	TcSAUR75	Thecc1EG029218	EOY27353.1	123	13.61	5.34	− 0.15	95.85
76	TcSAUR76	Thecc1EG029294	EOY27449.1	98	11.15	7.76	− 0.37	90.51
77	TcSAUR77	Thecc1EG029295	EOY27450.1	116	13.47	9.15	− 0.47	94.74
78	TcSAUR78	Thecc1EG032432	EOY13787.1	124	14.22	8.76	− 0.33	102.98
79	TcSAUR79	Thecc1EG032976	XP_007022464.1	147	16.76	9.30	− 0.29	93.40
80	TcSAUR80	Thecc1EG034196	EOY14979.1	150	17.12	9.82	− 0.28	83.13
81	TcSAUR81	Thecc1EG034201	XP_007017765.1	104	11.90	7.81	− 0.27	88.94
82	TcSAUR82	Thecc1EG034344	XP_017981107.1	117	13.37	8.98	− 0.29	85.81
83	TcSAUR83	Thecc1EG036780	XP_007011526.1	161	18.41	9.36	− 0.53	78.70
84	TcSAUR84	Thecc1EG037466	XP_007012549.1	119	13.41	5.01	− 0.39	91.68
85	TcSAUR85	Thecc1EG037470	XP_007012552.1	160	17.98	5.36	− 0.47	78.50
86	TcSAUR86	Thecc1EG040303	XP_007014781.1	100	11.18	8.74	− 0.04	98.50
87	TcSAUR87	Thecc1EG042136	EOY34459.1	132	14.75	6.07	− 0.57	73.86
88	TcSAUR88	Thecc1EG042137	EOY34460.1	130	14.76	6.91	− 0.44	81.08
89	TcSAUR89	Thecc1EG043181	EOY18685.1	133	15.29	8.39	0.07	96.69
90	TcSAUR90	Thecc1EG044403	XP_007010524.1	101	11.11	9.75	− 0.01	88.81

Previously, the SAUR family is being explored in various higher plant species, such as potato [12], tomato [12], watermelon [13], cotton [14], moso bamboo [15], poplar [16], grape [17], apple [18], coffea [19], Chinese white pear [20], melon [21], loquat [22], wax gourd [23], peanut [24], pineapple [25], foxtail millet [26], cucumber [27], and longan [28]. More specifically, 31 and 38 members of the SAUR families have been recorded in coffea and moso bamboo [15, 19]. Previous studies also revealed 52, 60, 62, and 68 SAUR proteins in pineapple, grape, cucumber, and wax gourd [17, 23, 25, 27]. Meanwhile, a total of 98, 105, and 116 putative SAUR proteins was found in apple, poplar and Chinese white pear [16, 20, 47]. Our comparisons suggested that the SAUR families in higher plant species were large groups, with greatly variable members.

### Analysis of the general features of TcSAUR proteins in cacao

To better comprehend the TcSAUR proteins, the physic-chemical parameters of each member of the TcSAUR family, such as protein length, pI, mW, AI and GRAVY scores were analyzed as previously described [9, 33–36]. The general properties of the TcSAUR proteins were then provided in Table 1. We found that the proteins of TcSAUR family were varied from 60 (TcSAUR36) to 180 residues (TcSAUR54) in length (Table 1). The estimated mW ranged from 6.56 to 20.35 kDa, and TcSAUR36 and TcSAUR54 had the lowest and highest mW values, respectively (Table 1). The predicted pI scores of the TcSAUR proteins were varied from 4.04 (TcSAUR36) to 10.60 (TcSAUR11) (Table 1). Among them, a majority of members of the TcSAUR, particularly 68 out of 90 members had pI scores greater than 7.00 (Table 1). Next, the AI scores of the TcSAUR proteins were found between 65.75 (TcSAUR03) and 107.97 (TcSAUR66) (Table 1). Finally, 80 out of 90 TcSAUR proteins were predicted to be hydrophilic because their GRAVY scores were minus, ranging from − 0.81 (TcSAUR02 and TcSAUR55) to − 0.01 (TcSAUR37 and TcSAUR41) (Table 1). Ten remaining TcSAUR proteins, including TcSAUR18, TcSAUR26, TcSAUR27, TcSAUR34, TcSAUR38, TcSAUR40, TcSAUR44, TcSAUR49, TcSAUR67, and TcSAUR89, had plus GRAVY scores (Table 1), suggested that they were hydrophobic.

Previously, the general features of the SAUR proteins in higher plant species were discussed [20]. For example, the pI scores of the SAUR proteins in Chinese white pear ranged from 5.10 to 10.28, of which 63 (out of 116) SAUR proteins shared pI scores greater than 7.00 [20]. The mW values of the SAUR proteins in Chinese white pear have been reported to vary greatly, with the minimum mW and maximum mW of 7.47 and 122.22 kDa, respectively [20]. Similarly, the protein sizes of the SAUR proteins in Chinese white pear ranged from 67 to 1090 residues, while all proteins were hydrophilic (GRAVY scores were minus) [20]. In foxtail millet, the SAUR proteins were varied from 8.21 to 39.49 kDa in mass [26]. Interestingly, most of the SAUR proteins were basic molecules (pI scores greater than 7.00), whereas only 17 members of the SAUR family were acidic proteins (pI scores less than 7.00) [26]. The AI scores of the SAUR proteins in foxtail millet were varied from 53.19 to 104.15 [26]. In cucumber, the SAUR proteins were varied in mW values from 9.47 to 86.25 kDa, while the pI scores of these proteins ranged from 4.77 to 10.38 [27]. The sizes of the SAUR proteins in cucumber were reported to be between 84 and 746 residues, while the GRAVY scores of these molecules were varied from -0.96 to 0.05 [27].

### Analysis of gene structures and phylogenetic tree of TcSAUR proteins in cacao

**Fig. 2:**
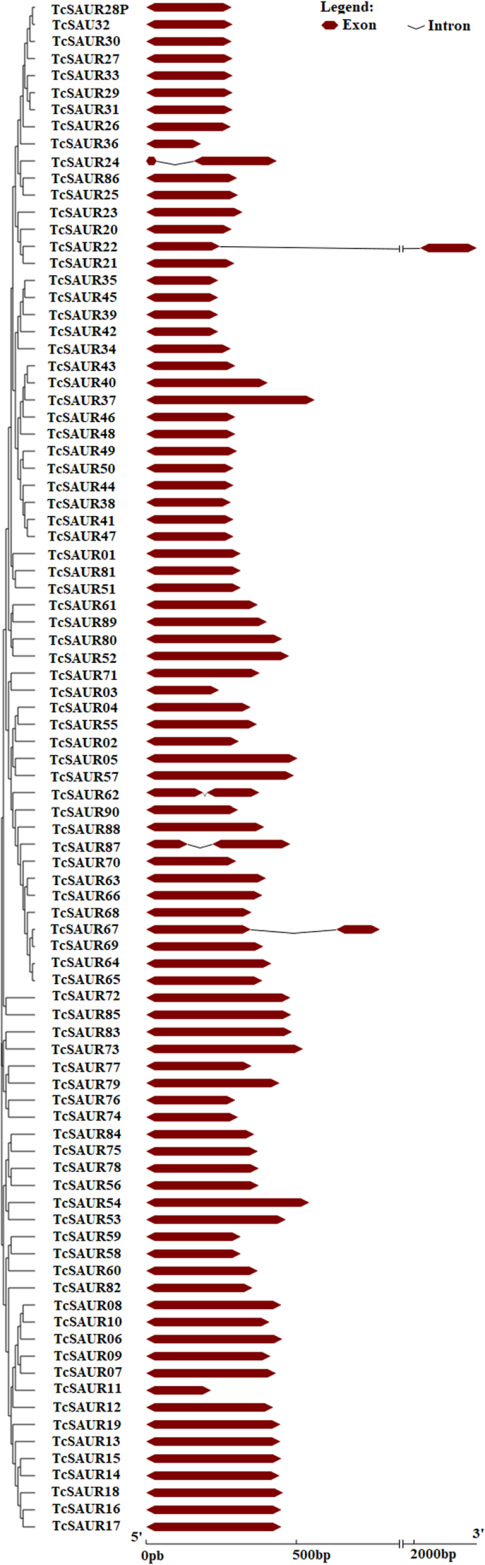
Exon/intron organization of the *SAUR* gene family in cacao

To get insight into the gene structures of the *TcSAUR* genes in cacao, we analyzed the exon/intron organization of all members. We found that 85 (out of 90) *TcSAUR* genes were intronless (Fig. 2). Five remaining *TcSAUR* genes, including *TcSAUR22*, *TcSAUR24*, *TcSAUR62*, *TcSAUR67*, and *TcSAUR87* contained two exons (Fig. 2). Additionally, the coding DNA sequences of the *TcSAUR* genes were varied from 183 (*TcSAUR36*) to 2209 nucleotides (*TcSAUR22*) (Fig. 2). The high occurrence of intronless genes in the *TcSAUR* family in cacao could be consistent with the cases reported in other plant species. For example, most *SAUR* genes in pineapple did not have introns [25], while 85 (out of 95) *SAUR* genes in loquat also contained no intron [22]. In Chinese white pear, a majority of the *SAUR* genes were intronless, whereas only five *SAUR* genes contained at least one intron [20]. Similarly, 94 (out of 105) *SAUR* genes in poplar contained no introns [16]. Taken together, our study suggested that most *SAUR* genes in cacao, perhaps in plant species did not have introns.

**Fig. 3:**
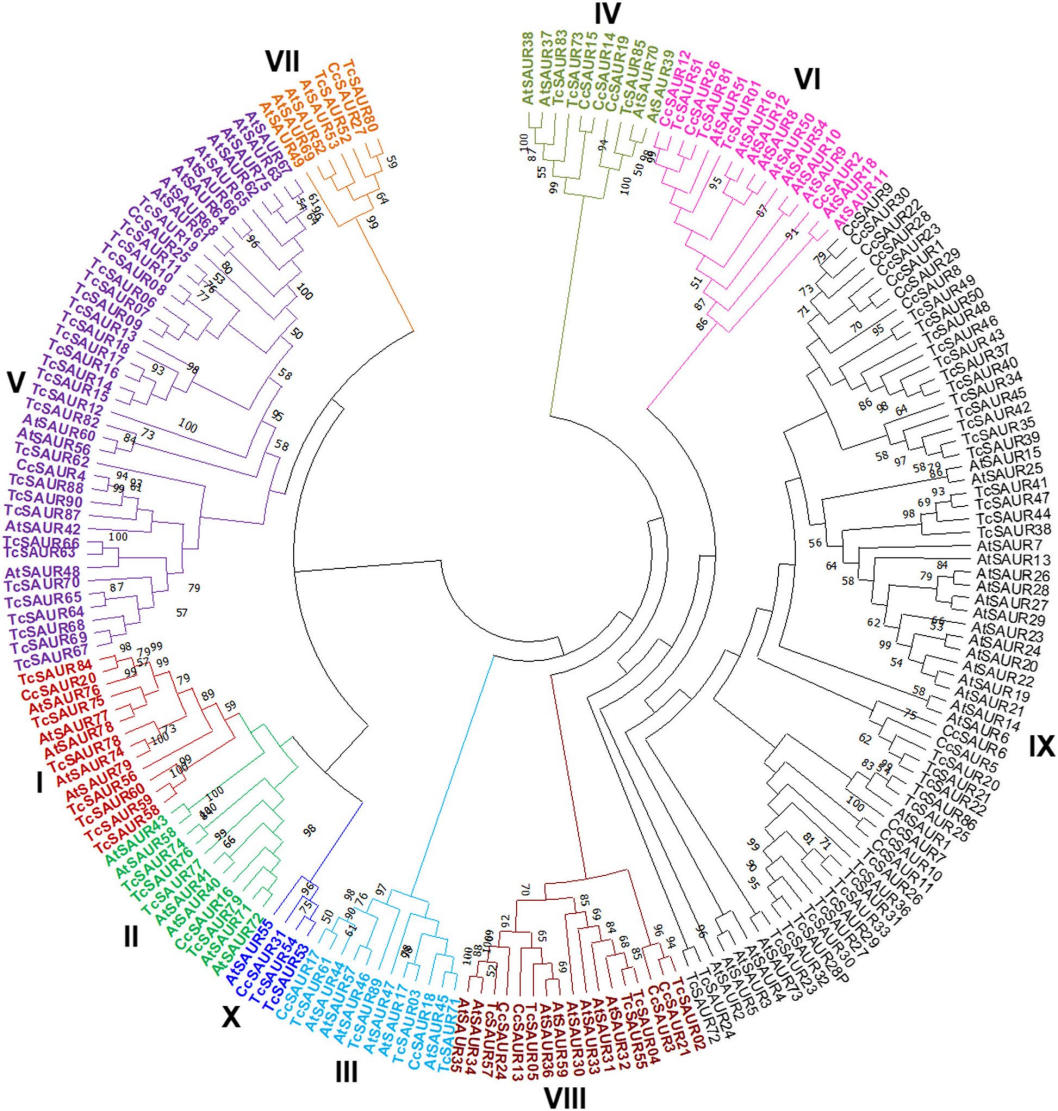
Categorization of the SAUR families in *Arabidopsis thaliana* (At), coffea (Cc), and cacao (Tc)

Next, to understand the relationship of the SAUR proteins, all members of the SAUR families from *Arabidopsis thaliana* [39, 40], coffea [19] and cacao were used to construct a maximum likelihood-based phylogenetic tree. As provided in Fig. 3, all SAUR proteins from *Arabidopsis thaliana* [39, 40], coffea [19], and cacao were clearly classified into seven clades. According to the phylogenetic tree, whole members of the TcSAUR family were distributed in all seven clades (Fig. 3). Particularly, seven clades could be assigned into 10 sub-groups. Among them, sub-groups 4 and 6 contained three (TcSAUR73, TcSAUR83, and TcSAUR85) and three (TcSAUR01, TcSAUR51, and TcSAUR81) members of the TcSAUR family, while only two (TcSAUR52 and TcSAUR80) and two (TcSAUR53 and TcSAUR54) members of the TcSAUR family have been found in sub-groups 7 and 10, respectively (Fig. 3). Sub-groups 9 and 5 shared the highest members of the TcSAUR family, with 33 and 27 TcSAUR proteins, respectively, while sub-groups 8, 3, and 2 harbored five (TcSAUR02, TcSAUR04, TcSAUR05, TcSAUR55, and TcSAUR57), four (TcSAUR03, TcSAUR61, TcSAUR71, and TcSAUR89), and four (TcSAUR74, TcSAUR76, TcSAUR77, and TcSAUR79) members (Fig. 3). Sub-group 1 had seven members, including TcSAUR56, TcSAUR58, TcSAUR59, TcSAUR60, TcSAUR75, TcSAUR78, and TcSAUR84 (Fig. 3). Previously, the SAUR families in cucumber and wax gourd were categorized into seven branches [23, 27]. Meanwhile, all 52 and 60 members of the SAUR family in pineapple and grape, respectively, were divided into 12 sub-families based on phylogenetic analysis [17, 25].

As a major part of this study, the duplication events that occurred in the *TcSAUR* gene family in cacao have been predicted. According to the classification of the duplicated genes [42], a total of four, three, and three duplication events resulting from tandem duplication, segmental duplication, and whole genome duplication have been found in the *TcSAUR* gene family in cacao (Table 2). Particularly, a large cluster of 13 and three *TcSAUR* genes, including *TcSAUR06*, *TcSAUR07*, *TcSAUR08*, *TcSAUR09*, *TcSAUR10*, *TcSAUR11*, *TcSAUR12*, *TcSAUR13*, *TcSAUR14*, *TcSAUR15*, *TcSAUR16*, *TcSAUR17*, and *TcSAUR18* and *TcSAUR20*, *TcSAUR21*, and *TcSAUR22*, were found as tandemly duplication events in chromosome 2 (Fig. 1, Table 2). One tandemly duplication event of seven *TcSAUR* genes (*TcSAUR63*, *TcSAUR64*, *TcSAUR65*, *TcSAUR66*, *TcSAUR67*, *TcSAUR68*, and *TcSAUR69*) localized in chromosome 5 was also reported (Fig. 1, Table 2). Next, three duplicated *TcSAUR* pairs were recorded as segmental duplication, including a pair of five genes (*TcSAUR26*, *TcSAUR29*, *TcSAUR31*, *TcSAUR33*, and *TcSAUR36*), a pair of 14 genes (*TcSAUR34*, *TcSAUR35*, *TcSAUR38*, *TcSAUR39*, *TcSAUR41*, *TcSAUR42*, *TcSAUR43*, *TcSAUR44*, *TcSAUR45*, *TcSAUR46*, *TcSAUR47*, *TcSAUR48*, *TcSAUR49*, and *TcSAUR50*), and a pair of *TcSAUR74* and *TcSAUR76* (Fig. 1, Table 2). Additionally, three gene pairs, including *TcSAUR04* (chromosome 1) and *TcSAUR55* (chromosome 3), *TcSAUR24* (chromosome 2) and *TcSAUR72* (chromosome 6), and *TcSAUR25* (chromosome 2) and *TcSAUR86* (chromosome 9) have resulted from the whole genome duplication events (Fig. 1, Table 2). Previously, tandemly duplication events, segmental duplication events, and whole genome duplication events were three main reasons for the expansion of the *SAUR* gene families in other crop species, like cotton [14], peanut [24], wax gourd [23], Chinese white pear [20], and foxtail millet [26].

**Table 2:** Duplication events found in the SAUR family in cacao

Duplication event	Duplicated genes
Tandem duplication	*TcSAUR06, TcSAUR07, TcSAUR09, TcSAUR08, TcSAUR10, TcSAUR11, TcSAUR12, TcSAUR13, TcSAUR18, TcSAUR14, TcSAUR15, TcSAUR16, TcSAUR17*
	*TcSAUR20, TcSAUR21, TcSAUR22*
	*TcSAUR53, TcSAUR54*
	*TcSAUR63, TcSAUR66, TcSAUR64, TcSAUR65, TcSAUR67, TcSAUR69, TcSAUR68*
Segmental duplication	*TcSAUR26, TcSAUR36, TcSAUR29, TcSAUR31, TcSAUR33*
	*TcSAUR34, TcSAUR45, TcSAUR42, TcSAUR35, TcSAUR39, TcSAUR38, TcSAUR44, TcSAUR41, TcSAUR47, TcSAUR49, TcSAUR50, TcSAUR48, TcSAUR46, TcSAUR43*
	*TcSAUR74, TcSAUR76*
Whole genome duplication	*TcSAUR04, TcSAUR55*
	*TcSAUR24, TcSAUR72*
	*TcSAUR25, TcSAUR86*

### Analysis of the TcSAUR genes expression profiles during the zygotic and somatic embryo maturation of cacao

**Fig. 4:**
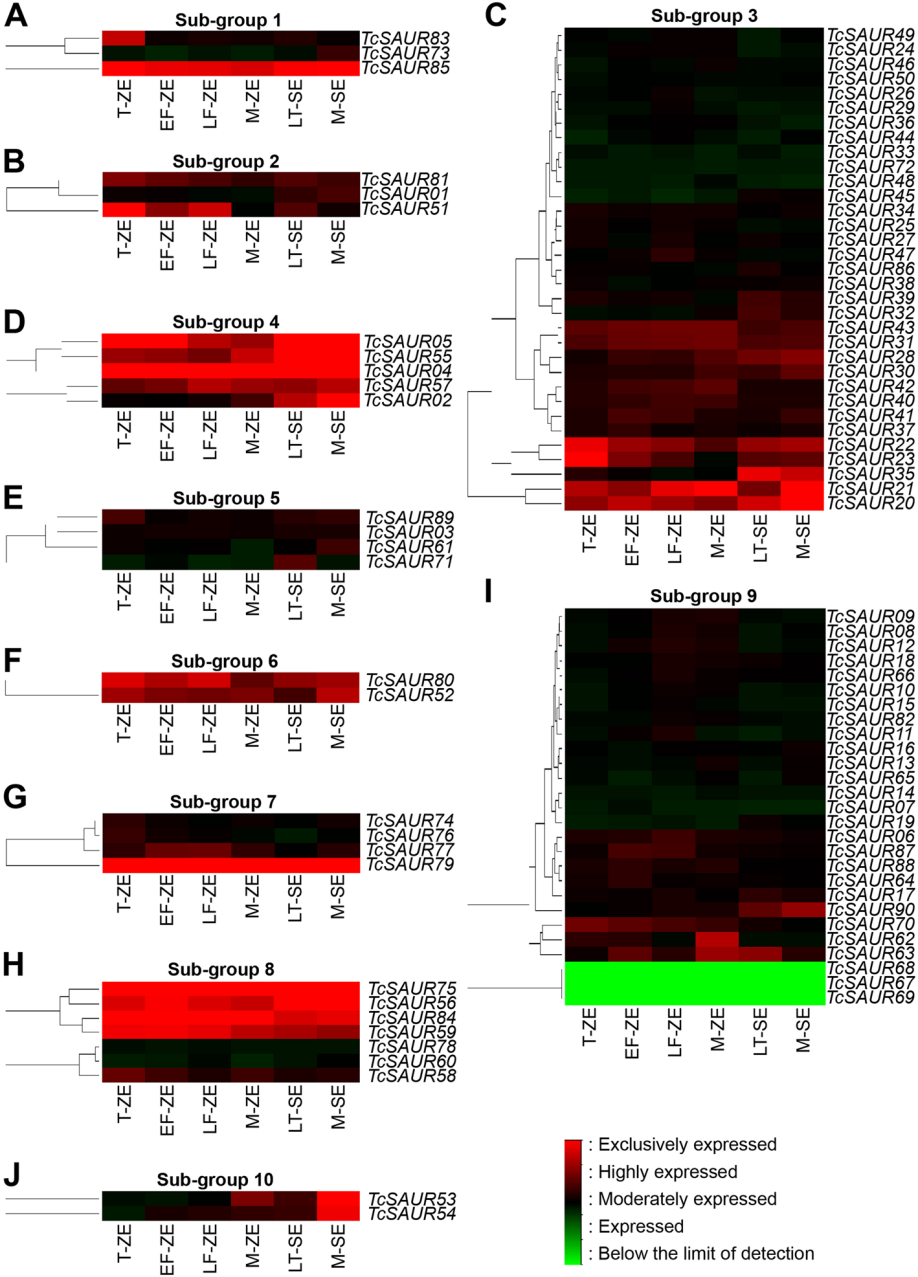
Expression patterns of the *SAUR* gene family during the zygotic embryogenesis and somatic embryogenesis in cacao

Of our interest, we investigated the expression patterns of the *TcSAUR* genes during the zygotic embryogenesis and somatic embryogenesis by re-explored the previous microarray data [45]. We then arranged the whole 90 members of the *TcSAUR* gene family into 10 sub-groups and provided in Fig. 4. As provided in Fig. 4, all *TcSAUR* genes were differentially expressed in six samples during the zygotic embryogenesis and somatic embryogenesis. Particularly, *TcSAUR85* was exclusively expressed in all six samples, while *TcSAUR83* tend to highly express in T-ZE (Fig. 4A). In sub-group 2, only *TcSAUR51* exhibited a strong expression during the zygotic embryogenesis (Fig. 4B). In sub-group 3, at least two genes, particularly *TcSAUR20* and *TcSAUR21*, were noted to be strongly expressed during the zygotic embryogenesis and somatic embryogenesis, while *TcSAUR35* was highly expressed in the LT-SE and M-SE (Fig. 4C). Two genes, like *TcSAUR22* and *TcSAUR23*, were exclusively expressed in T-ZE (Fig. 4C). Interestingly, a majority (four out of five) members of the *TcSAUR* family belonging to sub-group 4, including *TcSAUR04*, *TcSAUR05*, *TcSAUR55*, and *TcSAUR57*, exhibited a strong expression in both six tissues, whereas *TcSAUR02* was highly expressed in LT-SE and M-SE (Fig. 4D). We also found that *TcSAUR* genes in sub-group 5 tend to be moderately expressed in all tissues during zygotic embryogenesis and somatic embryogenesis (Fig. 4E). Additionally, two (*TcSAUR52* and *TcSAUR80*), one (*TcSAUR79*), and four (*TcSAUR56*, *TcSAUR59*, *TcSAUR75* and *TcSAUR84*) genes in sub-group 6, 7, and 8 were strongly expressed in all samples (Fig. 4F, G, H). In sub-group 9, *TcSAUR90* was highly expressed in LT-SE and M-SE, while TcSAUR62 and TcSAUR63 proteins were highly accumulated in M-ZE (Fig. 4I). Finally, two *TcSAUR* genes in sub-group 10, like *TcSAUR53* and *TcSAUR54*, were exclusively expressed in M-SE (Fig. 4J).

Up till now, the SAUR functions in higher plant species have been investigated. For example, a recent study found that a member of the SAUR in tomato, namely *SlSAUR69*, increased fruit sensitivity to ethylene by suppressing polar auxin transport to alter the unripening-to-ripening transition [12]. Previously, the functions of the *SAUR* genes during embryogenesis were also recorded. Specifically, a number of the *SAUR* genes in coffea exhibited more expression in at least one of the developing embryo stages or plantlets [19]. Among them, the expression of coffea *SAUR12* gene increased in non-embryogenic calli and the developing embryo stages [19]. In coconut, the expression patterns of the *SAUR* genes in the embryogenic callus stage were reported to be significantly higher than that in the initial culture and somatic embryo stage [48]. Recently, a number of the *SAUR* genes in longan were strongly expressed in the globular embryos, suggesting that they might play an important role during the early longan somatic embryogenesis [28]. In the future, point-mutation genetic tests should be performed to confirm their crucial significance in the biochemical function of TcSAUR proteins in cacao.

## Conclusion

To sum up, this current study provided new insight into the identification, annotation, characterization, and expression of the *TcSAUR* gene family in cacao. Our results indicated that all members of the TcSAUR family were slightly conserved based on their structure and phylogenetic tree. Among them, our results clearly indicated that tandemly segmental duplication events, segmental duplication events, and whole genome duplication events could be explained for the evolution of this important gene family. Of our interest, we found that the expression of the *TcSAUR* genes showed significant expression levels in various tissues during the zygotic embryogenesis and somatic embryogenesis by re-analyzing the previous microarray database. Taken together, our study provided fundamental information on the molecular mechanism of *TcSAUR* genes involved in cacao embryogenesis. Manipulation of *TcSAUR* expression will facilitate and accelerate zygotic embryogenesis and somatic embryogenesis during cacao tissue culture.
